# Bacterial isolates from drinking water river sources exhibit multi-drug resistant trait

**DOI:** 10.1007/s10661-024-13117-9

**Published:** 2024-10-15

**Authors:** Bukola Margaret Popoola, Jemimah Pearl Ogwerel, Oluwatosin Gbemisola Oladipo

**Affiliations:** 1 Department of Microbiology and Biotechnology, Ajayi Crowther University, Oyo, Oyo State, Nigeria; 2 Department of Microbiology and Biotechnology, Faculty of Natural and Applied Sciences, First Technical University, Ibadan, Nigeria; 3 Unit for Environmental Sciences and Management, North-West University, Potchefstroom Campus, Private Bag X6001, Potchefstroom, 2520 South Africa

**Keywords:** Antibiotic-resistant bacteria, Antimicrobial resistance, Freshwater, Physicochemical parameters, Potable water, Public and human health

## Abstract

Freshwater habitat is a natural reservoir for antimicrobial resistance (AMR). AMR poses serious human, animal, and environmental public health threats. This study aimed to evaluate the physicochemical and microbiological quality of five selected rivers (Apitipiti 1, Apitipiti 2, Apitipiti 3, Sogidi, and Aba Apa Akinmorin) in Oyo town, Nigeria, as well as the antibiotic resistance pattern of isolated bacterial species, using conventional methods. Most physicochemical parameters were within WHO and NIS permissible limits. Pearson’s correlation matrix indicated that there were significant (*p* < 0.05) interactions among pH, electrical conductivity, temperature, sulphate and chloride salts, and BOD and COD. A total of thirty-two (32) bacterial species were isolated and identified as: *Aeromonas* (9)*, Bacillus* (2)*, Corynebacterium* (13)*, Lactobacillus* (1)*, Pseudomonas* (2)*, Staphylococcus* (4)*,* and *Streptococcus* (1). Of the rivers, Sogidi had the highest microbial load (6.36 log CFU/mL) while Apititipiti 1 had the lowest (5.76 log CFU/mL). With regard to antibiotic sensitivity, 81.8% were multidrug-resistant, with *Corynebacterium kutscheri* and *Aeromonas* spp. isolated from Apitipiti 2 and Aba Apa Akinmorin rivers, respectively, exhibiting a relatively high antibiotic resistance of 90.9%. This study reveals that these rivers may be unfit for consumption as multidrug-resistant bacteria of public health risk were associated with them.

**Supplementary Information:**

The online version contains supplementary material available at 10.1007/s10661-024-13117-9.

## Introduction

About 70% of the earth’s surface is covered with water either as freshwater, brackish water or as saline water (Sen, 2019). Water, an important earth resource, is very vital to humans, plants and animals. Man utilises this indispensable natural resource for various activities including domestic, industrial, agricultural and or recreational purposes. To the ecosystem, water serves as a habitat for aquatic organisms such as fish, crocodiles, snails, frogs, bacteria, fungi, protozoans, algae and viruses and these organisms interact with each other and form the aquatic ecosystem (Sen, 2019).

Potable water is crucial to human existence. Unfortunately, some people struggle to have access to safe water. The physical and chemical properties of water have great implications on its microbial quality and the occurrence of antibiotic-resistant bacteria (ARB). Most often, when the physical (colour, odour, temperature etc.) and chemical (pH, electrical conductivity, heavy metals, COD, DO etc.) parameters of water are above permissible limits, the microbial load of the water will be high with elevated ARB levels (Ho et al., 2021). The microbial quality of water largely determines its usefulness, especially for domestic usage. Globally, bacterial contamination of water brings about serious public health threats (Hile et al., 2023). Bacteria are one of the major pathogens responsible for waterborne diseases, and they have been implicated in many gastrointestinal outbreaks worldwide (Delgado-Gardea et al., 2016). Worldwide as a result of waterborne diseases, 525,000 children (< 5) come down with diarrhoea on a yearly basis with 117,000 deaths recorded in Nigeria alone (WHO, 2017; UNICEF, 2018).

However, despite the continuous efforts to improve water quality worldwide, waterborne disease outbreaks are still reported (Hile et al., 2023). Bacteria associated with waterborne diseases include *Escherichia coli*, *Legionella* spp., *Pseudomonas aeruginosa*, *Aeromonas* and *Mycobacterium* spp. (Stec et al., 2022). Other waterborne pathogens are usually obligate pathogens; they multiply only in an infected host. They include *Campylobacter, Salmonella, Shigella, Escherichia coli, Acinetobacter* spp., *Clostridium* spp., *Bacillus anthracis* and *Helicobacter pylori*. (Delgado-Gardea et al., 2016). When in the human hosts, these waterborne pathogens are usually eliminated using antibiotics.

For decades, antibiotics are the widely known effective antimicrobials used for treatment of infectious diseases while curbing the rate of morbidity and mortality (Chukwu et al., 2020). However, the misuse of antibiotics has led to the development of antimicrobial resistance. Several sources of antibiotic contamination into water bodies have been identified which include: surface runoff, hospital discharge, sewage and wastewater treatment effluents, municipal waste and animal wastewater effluents (Guo et al., 2018). When consumed, antibiotics are broken down partially in the body; consequently, a large amount is excreted in their normal form or as active metabolites through urine and faeces, and these get into wastewater treatment plants (Grenni et al., 2018). Unfortunately, wastewater treatment plants are not designed to eliminate antibiotics; hence, these antibiotics are eventually released into the environment including freshwater (Grenni et al., 2018) by erosion, sewage runoff or leaching (Wang et al., 2022).

The deposition of antibiotic residues into the environment leads to the development of antimicrobial resistance in some bacteria. These bacterial species are referred to as antibiotic-resistant bacteria (ARB) and they contain antibiotic resistance genes (ARGs) which are transferred horizontally or vertically to other bacteria (Peterson & Kaur, 2018). Related studies have also revealed that heavy metals do combine with antibiotics in compound pollution (Zhou et al., 2022). The synergistic selection pressure between heavy metals and antibiotics increases the population of co-resistant microorganisms in the environment, impairs microbial communities and the expression of genes in water bodies causing changes in the structure and diversity of natural microbial communities (Wang et al., 2023).

Heavy metals, even at very low concentrations, can alter the bacterial efflux pump system expression, thereby promoting cross-resistance to antibiotics and contributing to the development of multidrug-resistant bacterial species (Xu et al., 2022; Chukwu et al., 2023). Bacterial species become vulnerable to antibiotics due to persistent and continuous exposure to antibiotics; this increases bacterial selection pressure which in turn enhances the increased abundance of resistant bacterial strains (Danner et al., 2019). Antimicrobial resistance (AMR) has become a serious global issue, gaining top priority on the public health agenda as it leads to a reduction in treatment options for bacterial infections thereby reducing clinical efficacy while increasing treatment costs and mortality (Oladipo et al., 2019; Achi et al., 2021).

To the best of our knowledge, this study provides a comprehensive first-hand information/literature with evidence on the surface water quality of selected rivers in Southwestern Nigeria. The study therefore evaluated the physicochemical parameters, microbial population and diversity, their interrelationships and the detection of antibiotic-resistant bacteria isolated from selected rivers in Oyo town, Nigeria, which serve as a major source of water supply to residents around the areas.

## Materials and methods

### Sample collection sites

Five rivers were strategically sampled in a Local Government Areas (LGA) — Atiba LGA, Oyo State, Nigeria. The five rivers studied are flowing water bodies and serve as sources of water supply and tourism to residents around the areas. The rivers (Table 1) are namely: (Apitipiti 1, Apitipiti 2, Apitipiti 3), used for fishing, (Sogidi), tourism (Sogidi and Aba Apa Akinmorin), and drinking.

**Table 1:** Sampling areas of selected rivers with their GPS locations

S/N	Site name	GPS readings
1	Apitipiti 1	7°48′4512″ N, 3°58′3035″ E
2	Apitipiti 2	7°49′8256″ N, 3°56′1570″ E
3	Apitipiti 3	7°49′8071″ N, 3°56′9658″ E
4	Sogidi	7°49′7074″ N, 3°56′7363″ E
5	Aba Apa Akinmorin	7°47′1797″ N, 3°57′2335″ E

### Sample collection

Sampling sites were purposely selected and some principal rivers within Oyo town were selected. Triplicate water samples were collected during the dry season (February) at a depth of 20 cm. While plastic bottles were used to collect water samples for physicochemical analysis, sterile McCartney bottles were used to collect water samples for microbiological analysis. The plastic bottles were rinsed with the river water thrice and water was collected. The sterile McCartney bottles were held with sterile forceps and dipped into the water and the water was collected and capped immediately. The forceps were sterilised intermittently with 70% ethanol. The bottles were labelled appropriately and were transported to the laboratory for analyses in an ice pack.

### Water quality evaluation

The physical and chemical properties of the various water samples were determined. The details of the analyses are as follows:

#### Physical properties

##### pH

The pH of each water sample was determined in situ and recorded accordingly during the sampling exercise using a hand digital metre (HI 9812–5, Hanna Instruments, Woonsocket, Rhode Island, USA).

##### Temperature

Temperature readings for each water sample collected were obtained and recorded. A hand digital metre (HI 9812- 5, Hanna Instruments, Woonsocket, Rhode Island, USA) was used in situ.

##### Electrical conductivity

In addition, the electrical conductivity of each of the water sample collected was determined in situ using a hand digital metre (HI 9812–5, Hanna Instruments, Woonsocket, Rhode Island, USA). Each reading was recorded accordingly.

Chemical properties measured include heavy metal concentrations, biochemical oxygen demand (BOD), chemical oxygen demand (COD), dissolved oxygen (DO), turbidity and anions concentration.

### Chemical properties

#### Dissolved oxygen (DO)

The DO of the water samples collected was determined using the modified Winkler’s method (Ayandiran, 2014). Briefly, the sample was diluted using dilution water containing certain nutritive chemicals. The amount of dissolved oxygen present was then afterwards measured.

#### Biological oxygen demand (BOD)

The BOD of collected water samples was determined using modified Winkler’s method as described by Ayandiran (2014). The sample was diluted using dilution water containing certain nutritive chemicals. The amount of dissolved oxygen present was initially measured; after, the water was incubated in the dark at 20 °C for 5 days. After a 5-day incubation period, the amount of dissolved oxygen present was then measured again. The BOD was calculated using the formula below:

BOD mg/l = DO_0_ − DO_1_.

where.

DO is the dissolved oxygen of the diluted solution after preparation.

D1 is the diluted solution after 5 days of incubation.

#### Chemical oxygen demand (COD)

The COD of the water samples obtained were determined spectrometrically at 600 nm using Jenway 6405 UV/VIS, UK spectrophotometer. The absorbance of Cr^3+^ formed was measured, when organic and other oxidisable materials in the water samples were oxidised by digestion with potassium dichromate (KCr₂O_7_), under acidic condition (H_2_SO_4_) (Li et al., 2018). The chemicals used were of analytical grade and products of Sigma Aldrich (USA).

#### Turbidity

The turbidity of the obtained water samples was determined using a calibrated turbidity meter (HI93703C, Hanna Instruments, Woonsocket, Rhode Island, USA), following the techniques of nephelometry, which measures the intensity of scattered light. Samples were collected in clean, airtight containers and allowed to settle before measurement was done. Turbidity was recorded in nephelometric turbidity units (NTU) to quantify the concentration of suspended particles and water quality assessment (Patil et al., 2012).

#### Sulphate concentration

Sulphate was determined using the turbidimetric method. It was measured by the nephelometric method in which the concentration of turbidity was measured against the known concentration of prepared sulphate solution (Patil et al., 2012).

#### Nitrate concentration

The water samples were analysed for nitrate levels by treating with Brucine in sulfuric acid which produced a yellow colouration. The concentration of nitrate nitrogen was then calculated based on the absorbance of the solution at 470 nm (Greweling & Peech, 1968).

#### Phosphate concentration

The phosphate concentrations of water samples were determined using the molybdenum blue method. Briefly, 5 ml of water was added into a 50 ml volumetric flask, and distilled water was added to bring up the volume to the 40 ml mark. Eight millilitres of ascorbic acid was then added and mixed thoroughly. The absorbance or optical density of the coloured solution was read and matched against a reagent blank at 882 nm after 30 mins. (Murphy & Riley, 1962).

#### Chloride concentration

The Mohr method was used to determine the concentration of chloride in the water samples collected. Silver nitrate was used for titration (normality, 0.0141) this corresponds to 1 ml of 0.0141 which equals 1 mg chloride in solution. The silver nitrate solution was standardised against standard chloride solution, prepared from sodium chloride (NaCl). Titration end point occurred when the chloride ions were precipitated (Ayandiran et al., 2014).

#### Heavy metals

The concentrations of fluoride ion (F) and heavy metals such as Iron (Fe), Copper (Cu), Zinc (Zn), Chromium (Cr), Cadmium (Cd), Lead (Pb), Nickel (Ni) and Manganese (Mn) were determined using AAS — atomic absorption spectrophotometer (Model 210/211 VGP Buck Scientific Atomic Absorption Spectrometer, East Norwalk, Connecticut, USA) (Ayandiran et al., 2014).

### Microbial analysis

For the microbial analyses, samples were serially diluted into five test tubes that were labelled appropriately. One millilitre each of the water samples was dispensed into sterile test tubes containing 9 ml sterile distilled water; a serial dilution of fivefolds was carried out. One millilitre of the 3rd and 5th dilutions was pipette into sterile petri dishes using pour plate method.

#### Heterotrophic count

Enumeration of total heterotrophic bacteria (not particularly fastidious) counts was determined using Nutrient Agar (NA, Oxoid Ltd., Basingstoke, UK). The prepared nutrient agar was allowed to cool to about 45 °C – 50 °C, the mouth of the flask was flamed, then about 15 ml of the agar medium was poured aseptically into the petri dishes containing the samples. The plates were rocked gently immediately after pouring the agar so the microorganisms could be evenly separated during growth. Solidification of the agar was followed by incubation of the plates invertedly at 37 °C for 24 h. Three plates of the agar without samples were poured to serve as control. Colonies were observed, counted and recorded after 24 h. Distinct colonies were then sub-cultured until pure cultures were obtained and transferred onto slant bottles containing freshly prepared agars.

#### Enumeration of lactose fermenters and non-lactose fermenters

MacConkey Agar (MAC, Oxoid Ltd., Basingstoke, UK), a differential and selective culture medium for bacteria, was used to selectively isolate Gram-negative and enteric bacteria and differentiate them based on lactose fermentation. Lactose fermenters turned red on MacConkey agar, and non-fermenters showed no change in colour.

The prepared MacConkey agar was allowed to cool to about 45 °C – 50 °C, the mouth of the flask was flamed, then about 15 ml of the agar medium was poured aseptically into the petri dishes containing the samples. Afterwards, the same procedure as with the heterotrophic counts was followed.

### Enumeration of the Enterobacteriaceae

Eosin Methylene Blue Agar (EMB, Oxoid Ltd., Basingstoke, UK), which is a more sensitive and stable medium compared to others for similar purposes, was used to some extent to inhibit the growth of Gram-positive bacteria and isolate enterobacteriaceae. Its purpose was also to differentiate between lactose fermenting and non-fermenting bacteria. Non-fermenters of lactose and sucrose resulted in colourless colonies on the agar plates.

The prepared EMB agar was allowed to cool to about 45 °C – 50 °C, the mouth of the flask was flamed, then about 15 ml of the agar medium was poured aseptically into the petri dishes containing the samples. Afterwards, the same procedure as with the heterotrophic counts was followed.

#### Determination of mannitol-fermenting staphylococci

A selective and differential medium — Mannitol agar (MA, Oxoid Ltd., Basingstoke, UK) — was used for the enumeration of mannitol-fermenting staphylococci. The medium contained the sugar alcohol mannitol and phenol red indicator, a pH indicator for detecting acid produced by mannitol-fermenting staphylococci. *Staphylococcus aureus* produced yellow colonies with yellow zones, whereas other coagulase-negative staphylococci produced small pink or red colonies with no colour change to the medium.

The prepared Mannitol agar was allowed to cool to about 45 °C – 50 °C, the mouth of the flask was flamed, then about 15 ml of the agar medium was poured aseptically into the petri dishes containing the samples. Afterwards, the same procedure as with the heterotrophic counts was followed.

These four media were used in order to mitigate possible biases in results obtained. All media were prepared according to manufacturers’ instructions. Individual colonies were purified and identified by morphological and biochemical techniques (Dubey & Masheshwari, 2004). For proper identification of bacterial isolates, the obtained biochemical characteristics of the isolates were further confirmed on the National Center for Biotechnology Information (NCBI) platform (https://www.ncbi.nlm.nih.gov/).

### Antibiotic susceptibility testing

The antibiotic sensitivity test was carried out for each of the isolates using the Kirby Bauer Disc Diffusion method according to the guidelines of the Clinical Laboratory Standards Institute (CLSI, 2023). Control experiments were set up to serve as quality control. The antibiotics used were Imipenem (10 µg), Meropenem (10 µg), Ceftazidime (30 µg), Ciprofloxacin (5 µg), Gentamicin (10 µg), Chloramphenicol (12.5 µg), Tetracycline (30 µg), Oxacillin (1 µg), Cotrimoxazole (25 µg), Augmentin (20 µg), Vancomycin (30 µg) and Erythromycin (15 µg). The inoculum was prepared for each bacterial isolate by adjusting the turbidity to 0.5 McFarland standard and spread on Mueller–Hinton agar plates. The antibiotic discs were placed on the agar plates and incubated overnight at 37 °C for 24 h. The zones of inhibition (ZOI) were measured, and the isolates were classified as sensitive, intermediate or resistant according to CLSI tables and guidelines (Bayot & Bragg, 2021).

### Statistical analysis

All statistical analysis of data obtained was done using one-way analysis of variance (ANOVA) at a 5% level of significance using the Statistical Package for Social Sciences (SPSS) version 28 (IBM, Armonk, NY, USA). A post hoc test was performed using Duncan’s New Multiple Range Test. Pearson’s correlation (*r*) and cluster (*r* = 0.15 and 0.40, *p* < 0.05) analyses were used to determine interrelationships between the parameters.

## Results and discussion

### Physicochemical characteristics of sampled rivers

Table 2 presents the physicochemical characteristics of the sampled rivers. The results obtained were compared with the acceptable levels of the World Health Organisation (WHO) standards and the Nigerian Institute Standard (NIS) for drinking water.

**Table 2:** Physicochemical analysis of the water samples from the selected rivers

Parameters	Water samples					WHO Standard (2007)	NIS (2011)
	A	B	C	D	E		
Temperature (°C)	36.00 ± 0.0^a^	33.00 ± 0.0^a^	34.00 ± 0.0^a^	29.00 ± 0.0^a^	29.00 ± 0.0^a^	NA	Ambient
pH	8.50 ± 0.0^a^	9.30 ± 0.0^a^	8.60 ± 0.0^a^	6.70 ± 0.0^a^	6.80 ± 0.0^a^	6.5 – 8.5	6.5 – 9.5
EC (µS/cm)	100.00 ± 0.58^a^	320.00 ± 0.58^d^	540.00 ± 0.58^e^	260.00 ± 0.58^c^	130.00 ± 0.58^b^	1000	1000
Iron (Mgl^−1^)	40.011 ± 0.0017^e^	1.411 ± 0.00058^c^	0.611 ± 0.00^b^	0.443 ± 0.00058^a^	4.679 ± 0.00058^d^	0.3	0.3
Copper (Mgl^−1^)	0.110 ± 0.00058^d^	0.074 ± 0.0^b^	0.051 ± 0.00058^a^	0.080 ± 0.0029^c^	0.760 ± 0.00^bc^	2.000	1.000
Zinc (Mgl^−1^)	0.037 ± 0.0017^c^	0.049 ± 0.0017^d^	0.246 ± 0.0017^e^	0.003 ± 0.00058^a^	0.029 ± 0.0017^b^	NA	3.000
Chromium (Mgl^−1^)	0 ± 0.0	0 ± 0.0	0 ± 0.0	0 ± 0.0	0 ± 0.0	0.05	0.05
Cadmium (Mgl^−1^)	0 ± 0.0	0 ± 0.0	0 ± 0.0	0 ± 0.0	0 ± 0.0	0.003	0.003
Lead (Mgl^−1^)	0.01 ± 0.0^c^	0.0 ± 0.0^a^	0.003 ± 0.00058^b^	0.003 ± 0.00058^b^	0.002 ± 0.00058^b^	0.01	0.01
Nickel (Mgl^−1^)	0.0 ± 0.0^a^	0.0 ± 0.0^a^	0.068 ± 0.02^c^	0.041 ± 0.001^b^	0.014 ± 0.0^a^	2.0	1.0
Chloride (NTU)	43.20 ± 0.551^a^	64.80 ± 2.354^c^	93.27 ± 0.343^d^	50.40 ± 0.064^b^	43.20 ± 0.029^a^	250	250
Sulphate (Mgl^−1^)	3.355 ± 0.0^c^	3.375 ± 0.00058^d^	2.4280 ± 0.0055^b^	0.0 ± 0.0^a^	0.0 ± 0.0^a^	500	100
Phosphate (Mgl^−1^)	0.404 ± 0.002^b^	0.209 ± 0.001^a^	0.638 ± 0.013^c^	0.204 ± 0.0^a^	0.200 ± 0.0003^a^	1.0	1.0
Turbidity (Mgl^−1^)	8.533 ± 0.694^d^	7.033 ± 0.186^c^	5.967 ± 0.088^bc^	5.0 ± 0.231^ab^	4.0 ± 0.058^a^	5	5
Nitrate (Mgl^−1^)	0.0039 ± 0.00009^b^	0.0 ± 0.0^a^	0.0 ± 0.0^a^	0.316 ± 0.0013^d^	0.046 ± 0.00007^c^	50	50
COD (Mgl^−1^)	64.67 ± 0.333^e^	24.33 ± 2.028^b^	8.0 ± 0.577^a^	32.33 ± 0.333^c^	40.0 ± 1.732^d^	10	NA
BOD (Mgl^−1^)	5.17 ± 0.088^b^	2.60 ± 0.346^a^	3.13 ± 0.606^a^	2.37 ± 0.745^a^	2.93 ± 0.606^a^	5.0	NA
Manganese (Mgl^−1^)	0.20 ± 0.0115^c^	0.06 ± 0.0058^b^	0.10 ± 0.0^a^	0.02 ± 0.0058^a^	0.083 ± 0.0088^b^	0.4	NA
Fluoride (Mgl^−1^)	0.110 ± 0.0058^c^	0.06 ± 0.0115^b^	0.037 ± 0.0088^ab^	0.01 ± 0.0^a^	0.04 ± 0.0115^b^	1.5	NA

*WHO* World Health Organisation, *NIS* Nigerian Industrial Standard, *NA* Not available.

Key: A — Apitipiti 1; B — Apitipiti 2; C — Apitipiti 3; D — Sogidi; E — Aba Apa Akinmorin; COD — chemical oxygen demand; BOD — biological oxygen demand; EC — electrical conductivity.

#### pH and temperature variations in sampled rivers

The pH of the water samples varied from river to river. Samples obtained from rivers Apitipiti 1, Sogidi and Aba Apa Akinmorin had pH values within WHO acceptable range (6.5 – 8.5). This finding correlates with the study of Ayandiran et al. (2014), with river water sampled being within WHO and NIS permissible ranges. However, Apitipiti 2 recorded slightly higher value of 8.6 while Apitipiti 3 (9.5) exceeded the WHO limit. Although, with reference to NIS, these river samples were within the permissible range (6.5 – 9.5), indicating that the water samples had acceptable pH values. A study by UMA (2016), however, observed that pH values above 6.5 – 8.0 negatively affect aquatic animals in rivers. Furthermore, in this study, the average pH recorded across the five rivers during the dry season of sampling was 7.98. Specifically, we observed a dearth in literature on the effect of pH on microbial load and antimicrobial resistance in surface water bodies. However, general studies on the influence of pH on bacterial population, diversity and antibiotic resistance revealed that low pH (3 to 5) significantly reduced bacterial population, diversity, activities and eliminated antibiotic-resistant bacteria (Yu et al., 2023). Furthermore, Gama et al. (2023) reported that higher pH values increased antibiotic resistance, bacterial diversity and population.

For the temperatures, there were no significant (*p* > 0.05) differences among the sampled rivers in this study. In addition, the recorded temperature ranges (29.0 – 36.0 °C) of all the river sites indicated a favourable environmental condition which supported the mesophilic bacteria isolated from the rivers. According to Hoorzook et al. (2021), the temperature of water is an essential environmental factor in aquatic ecosystems that affects the rate of metabolic activities of microorganisms. With regard to antibiotic resistance, Bagra et al. (2024) observed that temperature plays a key role and that increased temperatures in water bodies led to higher bacterial antibiotic resistance.

#### Electrical conductivity and soluble salts in river water samples

In this study, it was observed that the electrical conductivity (EC) and salt levels (chloride, fluorine, sulphate, phosphate and nitrate) of all the samples were within the WHO and NIS permissible limits. Although, the electrical conductivity (100 – 540 µS/cm) significantly (*p* < 0.05) varied from one river to another, all the values were within the WHO and NIS permissible limit (1000 µS/cm). While Apitipiti 1 recorded the least (100 µS/cm) EC, Apitipiti 3, had the highest value with 540 µS/cm. These findings correlate with the study of Hoorzook et al. (2021) where sampled rivers in South Africa, recorded EC values within the permissible limit. Low electrical conductivity level has been found to reduce levels of dissolved salts and hence decrease bacterial load (Ateba et al., 2020).

Similarly, salts of chloride, fluorine, sulphate, phosphate and nitrate levels of all the sampled rivers recorded values within the WHO and NIS permissible limits. This is in conformity with the findings of Egwuonwu et al. (2012), where the mineral content of the water samples studied was within the WHO standard.

This similar trend recorded for EC and the soluble salts can be ascribed to the direct interrelationship that exists between these parameters. In addition, Shoeb et al. (2022) reported that the level of electrical conductivity in water also indicated the presence of soluble ions. According to Cui et al. (2020), bacterial diversity is unaffected by water salinity variations due to their remarkable genetic plasticity and salt resistance mechanisms. Hence, bacterial abundance may not be influenced despite changes in salinity levels. Likewise, with antibiotic resistance, high salinity level has been linked to inhibition in growth of some bacteria carrying ARGs (Liu et al., 2018).

#### Heavy metal contents in sampled river waters

About 87.5% (7 out of 8) of the metals tested in the river water samples were within the WHO and NIS permissible limits. Only the iron concentrations in all analysed water samples exceeded both WHO and NIS permissible limit (0.3 Mgl^−1^) with Apitipiti 1 (40.011 Mgl^−1^) being significantly (*p* < 0.05) higher than all the others. This may be ascribed to the ongoing road construction activity taking place at the time of sample collection.

In addition, during sampling, it was observed that Apitipiti 1 River was surrounded by rocks. This may be ascribed to the elevated Fe concentrations recorded at the sampling site. The high iron levels recorded across the rivers in this study correlate with that of Kumar et al. (2017), where all water samples contained elevated iron content higher than the permissible WHO. The rivers sampled in this study may be considered unfit as a result of the elevated iron content. Many factors may have contributed to the high Fe levels of recorded across all the samples. According to Khatri et al. (2017), iron gains entry into water bodies via geogenic sources, industrial effluents and dumping of domestic waste or pollution from iron and steel industries, iron metal corrosion and iron mining. High iron intake in humans has been linked to haemochromatosis (Kumar et al., 2017), retinitis, conjunctivitis, choroiditis, cancer and heart diseases (Khatri et al., 2017).

Furthermore, for Cu (0.051 – 0.11 Mgl^−1^) and Zn (0.003 – 0.245 Mgl^−1^) contents in all the water samples, values recorded were within the WHO and NIS permissible range (1, 2 and 3 Mgl^−1^). According to Dietrich et al. (2004), surface and groundwater usually contain low concentrations of copper. This finding correlates with that of Kusmana et al. (2018), where the sampled rivers in Indonesia had copper concentrations below the WHO permissible ranges. However, a more recent study by Khatib et al. (2023) obtained elevated levels of Cu in river samples which is at variance with the trend recorded in this study. For Zn concentrations, this result conforms with the findings of Khatib et al. (2023), which obtained similar critically low Zn values from sampled river waters in Japan.

Heavy metals such as chromium (0.00 Mgl^−1^), cadmium (0.00 Mgl^−1^), lead (0.00 – 0.01 Mgl^−1^), manganese (0.02 – 0.2 Mgl^−1^) and nickel (< 0.2 Mgl^−1^) were all within the WHO and NIS permissible range. This could indicate that these water samples were free from some major anthropogenic activities such as solid mineral mining and smelting which could trigger health hazards. Elevated levels of metal in water have been identified to pose both human health and environmental threats (Senze et al., 2022). Human diseases such as cardiovascular disorders, neuronal damage, renal injuries and cancer have been reported as a result of heavy metal pollution (Rehman et al., 2018).

Generally, heavy metals are established co-pollutants with antibiotics in water bodies. Their presence in water sources has been ascribed to increased proliferation of antibiotic-resistant bacteria. According to Haberecht et al. (2019) and Mercimek Takcı and Toplar (2023), the presence of heavy metals may cause co-selection on the propagation of ARGs in aquatic environments. Studies have also revealed that heavy metals are the key factors influencing ARG contamination (Devarajan et al., 2015; Xu et al., 2017). Further, the dissemination of ARGs among vulnerable bacterial strains can lead to the emergence of entirely new ARB populations (Parvez and Khan, 2018).

#### Turbidity, chemical oxygen demand and biological oxygen demand

With regard to the turbidity levels of the five rivers sampled in this study, only one river - Aba Apa Akinmorin River recorded a value (4.0. Mgl^−1^) below the WHO and NIS of 5 Mgl^−1^ permissible limit. Apitipiti 1, 2 and 3 had turbidity values (8.533, 7.033 and 5.967 Mgl^−1^, respectively) that exceeded the permissible limit, while Sogidi River recorded the same concentration as the permissible limit. A similar study on river turbidity levels by Dongsheng et al. (2022) recorded the same trend as observed in this study. The implication of high turbidity on water bodies is high microbial load, reduction in the aesthetic value and quality of such rivers, subsequently having a negative impact on drinking, tourism and recreation.

The biological oxygen demand (BOD) of Apitipiti 2, Apitipiti 3, Sogidi and Aba Apa Akinmorin were below the permissible limit, while that of Apitipiti 1 was slightly above the permissible limit by 0.2 Mgl^−1^. The chemical oxygen demand (COD) of Apitipiti 1 (64.67 Mgl^−1^), Apitipiti 2 (24.33 Mgl^−1^), Sogidi (32.33 Mgl^−1^) and Aba Apa Akinmorin (40.0 Mgl^−1^) were way beyond the permissible limit (10 Mgl^−1^). However, Apitipiti 3 recorded a COD level below the permissible limit. From the results, water samples from Apitipiti 1 had both its BOD and COD values being significantly (*p* < 0.05) higher than all the other four rivers. The high COD as well as BOD values indicate an abundance of pollutants in the water. A similar study conducted by Zhao et al. (2020) observed this same trend in sampled river sources. BOD is the measure of the amount of utilised oxygen by microbes. Hence, the implication of a high BOD suggests the possibility of a high microbial load. This could be ascribed to probable pollution with improper disposal of domestic wastes, organic matter deposition such as faecal contamination and runoffs.

### Bacterial isolate counts and diversity from Atiba sampled rivers

Four media, namely, Nutrient Agar, MacConkey Agar, Eosin Methylene Blue Agar and Mannitol agar, were used to determine the bacterial heterotrophic counts and diversity represented in the five sampled rivers in this study.

### *Bacteria* total heterotrophic counts in sampled rivers

**Fig. 1:**
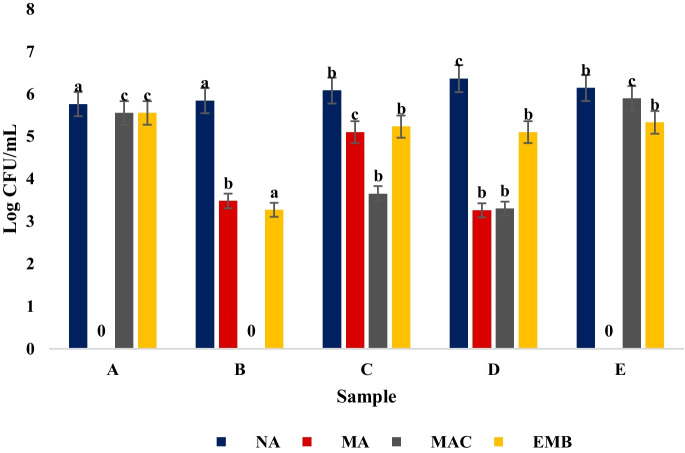
Heterotrophic bacterial plate counts from sampled rivers using different culture media. Key: Nutrient Agar, NA; MacConkey Agar, MAC; Eosin Methylene Blue Agar, EMB; and Mannitol agar, MA. A, Apitipiti 1; B, Apitipiti 2; C, Apitipiti 3; D, Sogidi; E, Aba Apa Akinmorin

The total heterotrophic bacterial counts in samples from Sogidi River had significantly (*p* < 0.05) higher microbial load (6.36 Log CFU/mL) compared to that of the other rivers (Fig. 1). Further, Staphylococcal species from Apititipiti 1 and Aba Apa Akinmorin were significantly (*p* > 0.05) lower (0.00 Log CFU/mL) compared to the other rivers. However, Gram-negative bacterial counts from Aba Apa Akinmorin River indicated significantly (*p* < 0.05) higher (5.90 Log CFU/mL) microbial load than the other rivers. The coliform counts from River Apititipiti 1 had significantly (*p* < 0.05) higher (5.78 Log CFU/mL) microbial load compared to others.

Overall, the total bacterial heterotrophic plate counts of all the water samples from the five rivers exceeded the WHO permissible limit for potable water (500 Cfu/mL). This indicates the rivers’ poor water quality, suggesting that they are unfit for drinking or domestic purposes. Similar findings by Onuoha (2017) and Ayandele et al. (2019) recorded high microbial load in river bodies. Generally, physicochemical (pH, temperature, EC, heavy metal content etc.) properties of water influence its microbial quality (Oladipo et al., 2019). Ho et al. (2021) established that other physicochemical properties such as DO determines to a great extent the microbial quality of water bodies. In particular, the high microbial load recorded from the rivers sampled in this study can be attributed to the elevated levels of physicochemical properties such as pH, turbidity, COD, BOD and heavy metal concentrations recorded.

#### Diversity of bacterial species in water samples of rivers

A total of thirty-two (32) bacterial species were isolated from the five sampling rivers using different culture media. Twenty-one (21) of the isolates were Gram-positive while eleven (11) were Gram-negative. This study does not follow the trend of some previous studies. For instance, Onuoha, (2017) found only 7 Gram-positive bacteria and 19 Gram-negative bacteria being isolated from different water sources in Southeastern Nigeria. In addition, another study conducted in Ghana by Odonkor et al. (2022) isolated more Gram-negative bacteria (83) compared to Gram-positive bacteria (27) from different water sources.

**Fig. 2:**
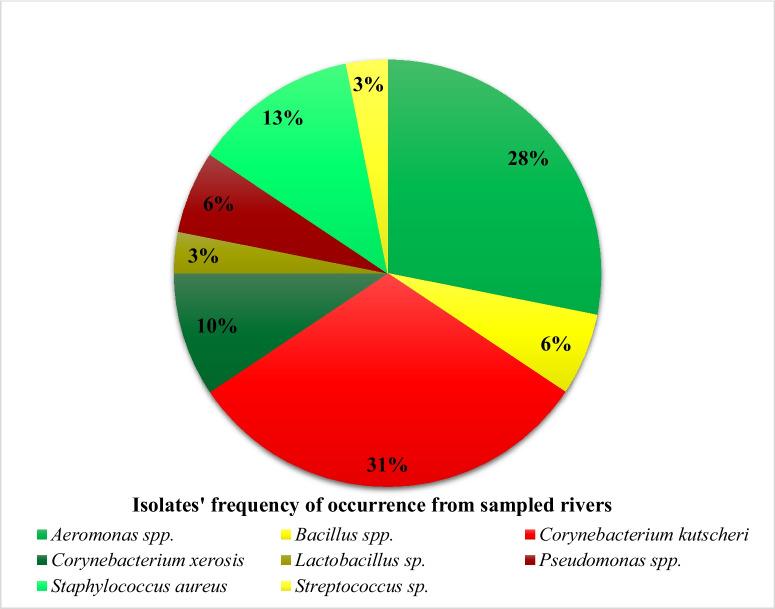
Diversity of occurrence of bacterial isolates isolated from rivers

In all, eight different genera (Fig. 2) of bacteria were isolated and identified as *Aeromonas* (9)*, Bacillus* (2), *Corynebacterium* (13), *Lactobacillus* (1), *Pseudomonas* (2), *Staphylococcus* (4) and *Streptococcus* (1). A study carried out by Ayandele et al. (2019) on a close-by dam to where this study was conducted isolated similar bacterial species of *Corynebacterium kutscheri, Streptococcus pyogenes, Shigella flexneri*, *Pseudomonas aeruginosa, Staphylococcus aureus* and *Escherichia coli*. Furthermore, *Aeromonas* were already isolated from sources like freshwater (Pessoa et al., 2022).

Although *Escherichia coli* was not detected in any of the five rivers sampled, this fact does not depict the complete absence of the organisms or other enteric bacteria. This finding correlates with the findings of Odonkor and Addo (2018) in a study conducted in Ghana, where no *E. coli* strain was isolated from river water sources. However, with the high levels of observed BOD and COD, this implies the possible presence of faecal contamination which probably was not just detected in the water at the time of sampling and calls for public health concerns.

*Corynebacterium kutsceri* had the highest frequency of occurrence of 11 while *Aeromonas* spp. followed closely with the second-highest frequency (9). *Lactobacillus* and *Streptococcus* had the lowest frequency of occurrence at (3.0%) (Fig. 2). It is worth noting that some *Aeromonas* spp. have been identified as pathogenic microbe associated with water bodies and its presence in the rivers has been implicated as a major public health concern (Pessoa et al., 2022).

### Relationships among parameters using correlation coefficient and cluster analyses

This study determined the correlation between different determined physicochemical, heavy metals and microbial parameters with the aid of correlation coefficient and cluster analyses.

#### Interrelationship between physical, chemical and biological parameters

For this study, the Pearson correlation analysis conducted indicated that there were significant relationships between the physicochemical and total heterotrophic counts variables. Sulphate exhibited significant to highly significant relationships with four key parameters (Supplementary Table 1). This was reflected with temperature (*r* = 0.93; *p* < 0.05), turbidity (*r* = 0.90; *p* < 0.05) and pH (*r* = 0.96; *p* < 0.01) having positive relationships and THB (*r* =  − 0.90; *p* < 0.05) which had a negative significant relationship. Temperature and turbidity (*r* = 0.91; *p* < 0.05) showed a significant relationship with one another. With regard to EC, there was a highly significant relationship with chloride (*r* = 0.98; *p* < 0.01) but a negative significant relationship with COD (*r* =  − 0.92; *p* < 0.05) was obtained. In addition, chloride showed a significant negative relationship with COD (*r* =  − 0.86; *p* < 0.05), while fluoride showed a significant positive relationship with BOD (*r* = 0.89; *p* < 0.05) and a negative significant correlation with THB (*r* =  − 0.92; *p* < 0.05). These findings further confirm the initial interrelationship observed among the physicochemical parameter and the bacterial counts. This result establishes that elevated levels of turbidity, BOD and COD are key factors that contributed significantly to the high microbial load and poor water quality observed in this study. This trend calls for public health concerns.

**Fig. 3:**
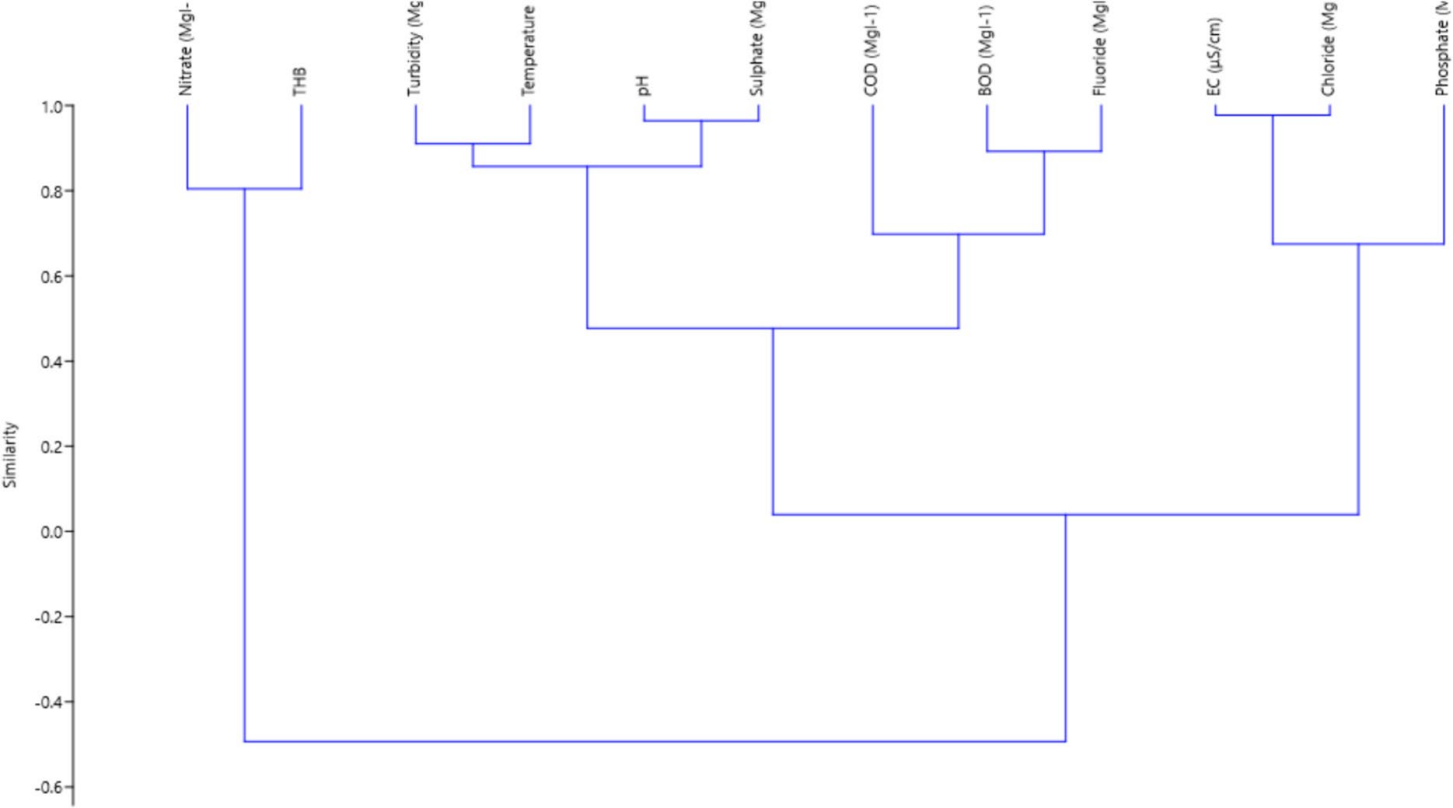
Hierarchical dendrogram of physicochemical, biological and microbial parameters obtained on river samples by the clustering method

Furthermore, the cluster analysis (Fig. 3) at 0.4, confirmed the trend observed in Supplementary Table 1. Three clusters were formed; the first cluster included nitrate and THB; the second cluster included turbidity, temperatures, pH, sulphate, COD, BOD and fluoride, while the third cluster revealed that similarity existed among EC, chloride and phosphate. These results indicate the interrelationship that exists among physicochemical and biological parameters. Previous studies (Saalidong et al., 2022) have established these findings. These deductions further confirm the likely public health challenges associated with these findings since the rivers are sources of potable water to the populace in those areas.

#### Interrelationship between heavy metals and total heterotrophic bacterial counts

**Fig. 4:**
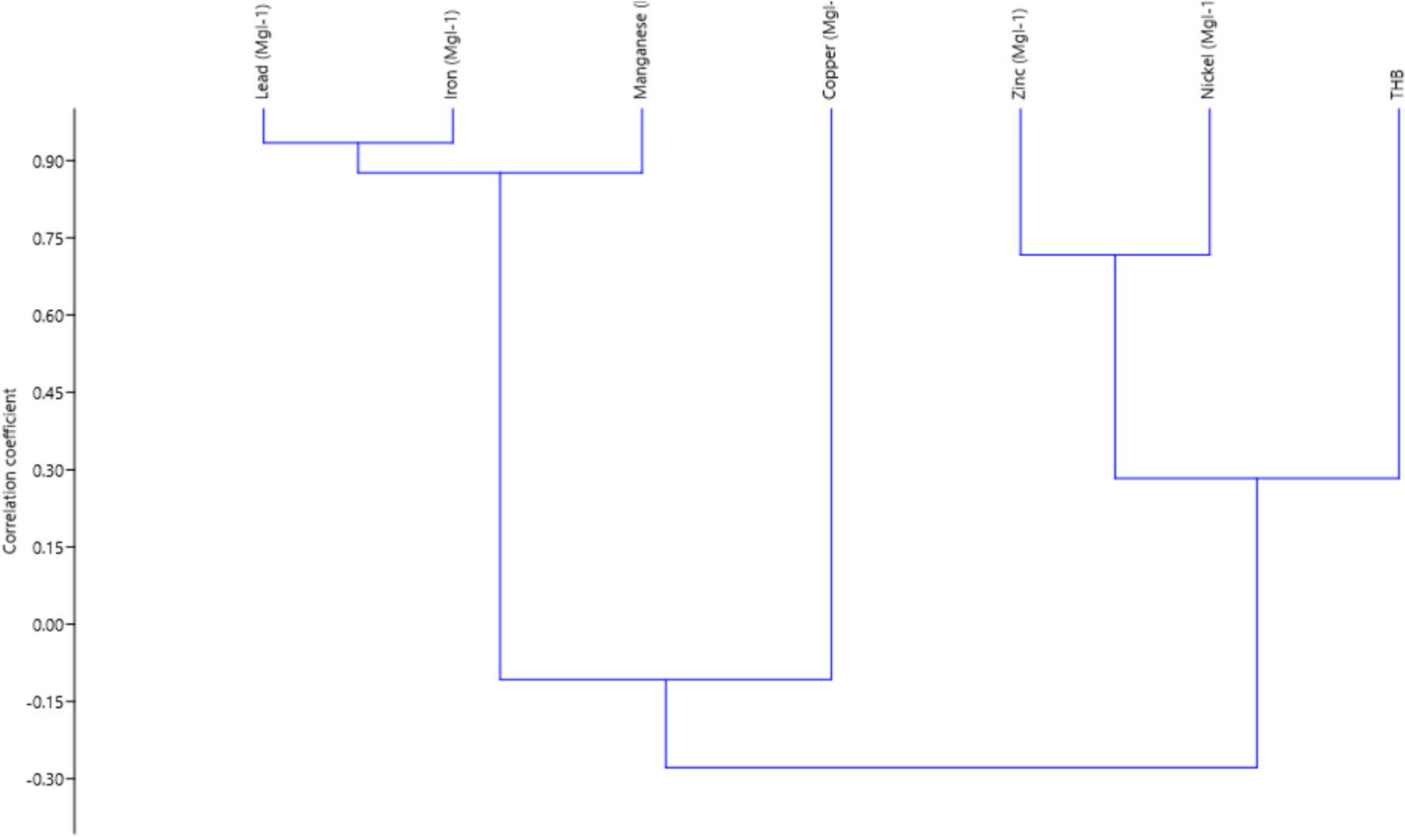
Cluster analysis of heavy metals and total bacterial counts in rivers sampled

For correlation analysis, relationship was only revealed between selected heavy metals but not with THB (Supplementary Table 2). Iron revealed a significant relationship with lead (*r* = 0.93; *p* < 0.05) and with manganese (*r* = 0.91; *p* < 0.05). Whereas when hierarchical cluster analysis was performed to ascertain the relatively homogeneous groups of heavy metals and their inter-relationship with bacterial counts (Fig. 4), aside, the first cluster formed at 0.15, confirming strong positive interactions among lead, iron and manganese and a second cluster formed with only copper, a third cluster indicating a very weak interaction among zinc, nickel and THB which linked with other heavy metals at 0.30 was also formed. This result confirmed strong positive interactions among lead, iron and manganese. A second cluster was formed with only copper, while a third cluster included zinc, nickel and THB which linked with other heavy metals at 0.30. A similar study by Trueman et al. (2019) corroborates the findings of this study that positive interactions exist among heavy metals. This further confirms that positive interactions exist among heavy metals and with bacterial abundance and diversity in water body sources.

### Exposure of isolated bacterial species from rivers to antibiotics

**Fig. 5:**
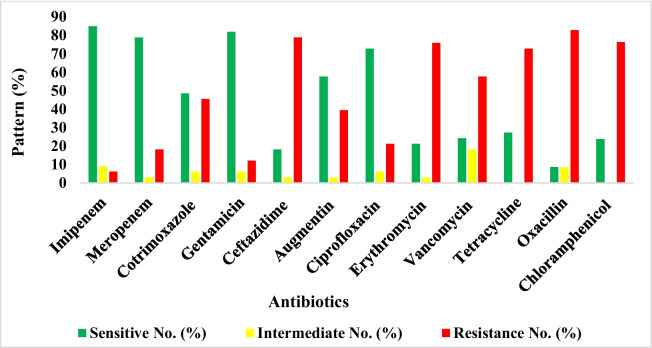
Antibiotic sensitivity pattern of the Gram-positive and Gram-negative bacterial isolates from the selected rivers

Bacterial species isolated from the five rivers when subjected to antibiotic susceptibility testing revealed that most of the bacterial isolates were susceptible to Carbapenems (Imipenem and Meropenem) and Gentamicin (Fig. 5). Although, on exposure to Ceftazidime and Oxacillin, most isolates exhibited resistance.

Of all the bacterial species isolated from the rivers sampled in this study, 81.8% showed multidrug resistance (MDR) with a multi-antibiotic resistance (MAR) index > 0.2 (Tables 3 and 4). Several studies have previously reported similar trends. From Africa, Ayandiran et al. (2014), in a study conducted in another Southwestern State in Nigeria, also reported a prevalence of 100% MDR bacteria; Addae-Nuku et al. (2022) also reported a prevalence of 55.5% MDR bacteria isolates from a river in Accra, Ghana. Furthermore, a study carried out by Koumaré et al. (2022) in Mali on the Niger River reported a prevalence of 81.8% MDR bacteria, being resistant to more than two antibiotics. In addition, other studies conducted in Malaysia by Salikan et al. (2020) on different rivers recorded a prevalence of 50% and 95.8% MDR bacteria, while in Spain, Perez-Etayo et al. (2020) reported 96.4% of isolates exhibited multi-drug resistance in some Northern Rivers.

**Table 3:** Antibiotic resistance profile of Gram-positive bacterial isolates from selected rivers

River source	Bacterial isolates	Resistance pattern	MAR Index	Resistance (%)
A	*Corynebacterium kutsceri*	OXA, CAZ, ERY, CHL	0.4	36.4
	*Corynebacterium kutsceri*	MEM, OXA, CAZ, CIP, ERY, VAN, CHL	0.5	54.5
	*Lactobacillus* sp.	-	0.0	0.0
	*Streptococcus* sp.	ERY, VAN, CHL	0.3	27.3
	*Staphylococcus aureus*	COT, OXA, CAZ, AUG, ERY, VAN, CHL	0.6	63.6
B	*Corynebacterium kutsceri*	OXA, CAZ, ERY, VAN, CHL	0.5	45.5
	*Staphylococcus aureus*	IMI, COT, OXA, CAZ, ERY, CHL	0.5	45.5
C	*Corynebacterium kutsceri*	MEM, COT, OXA, CAZ, AUG, ERY, VAN	0.6	63.6
	*Corynebacterium kutsceri*	COT, OXA, CAZ, AUG, CIP, ERY, VAN, CHL	0.7	72.7
	*Corynebacterium kutsceri*	OXA, CAZ	0.2	18.2
	*Bacillus* sp.	OXA, CAZ, ERY	0.3	27.3
	*Corynebacterium xerosis*	CAZ, CHL	0.2	18.2
	*Corynebacterium kutsceri*	IMI, MEM, COT, OXA, CAZ, AUG, ERY, VAN, CHL	0.6	63.6
	*Corynebacterium kutsceri*	CHL	0.1	9.1
	*Staphylococcus aureus*	COT, OXA, CAZ, AUG, CIP, ERY, VAN, CHL	0.7	72.7
	*Corynebacterium kutsceri*	OXA, CAZ, ERY	0.3	27.3
	*Corynebacterium kutsceri*	OXA, CAZ, CHL	0.3	27.3
	*Corynebacterium kutsceri*	IMI, MEM, COT, OXA, CAZ, AUG, CIP, ERY, VAN, CHL	0.9	90.9
D	*Bacillus* sp.	CAZ, ERY, CHL	0.3	27.3
E	*Staphylococcus aureus*	COT, OXA, CAZ, CIP, ERY, VAN, CHL	0.5	54.5
	*Corynebacterium xerosis*	CAZ, CHL	0.2	18.2

Key: A — Apitipiti 1; B — Apitipiti 2; C — Apitipiti 3; D — Sogidi; E — Aba Apa Akinmorin; R — resistant; I — intermediate, S — sensitive, and µ — microgram. IMI — Imipenem (10 µg); MEM — Meropenem (10 µg); COT — Cotrimoxazole (25 µg); GEN — Gentamicin (10 µg); CAZ — Ceftazidime (30 µg); AUG — Augmentin (20 µg); CIP — Ciprofloxacin (5 µg); ERY — Erythromycin (15 µg); VAN — Vancomycin (30 µg); OXA — Oxacillin (1 µg); CHL — Chloramphenicol (12.5 µg); MAR — multi antibiotics resistance.

**Table 4:** Antibiotics resistance profile of Gram-negative bacteria isolated from water samples of rivers studied

River source	Bacterial isolates	Resistance pattern	MAR Index	Resistance (%)
B	*Aeromonas* sp*.*	MEM, COT, OXA, CAZ, AUG, ERY, TET, VAN	0.7	72.7
	*Aeromonas* sp*.*	COT, GEN, OXA, AUG, CIP, ERY, TET, VAN	0.7	72.7
	*Aeromonas* sp*.*	MEM, COT, GEN, OXA, CAZ, AUG, CIP, ERY, TET, VAN	0.9	90.9
	*Aeromonas* sp*.*	OXA, CAZ, ERY	0.3	27.3
C	*Aeromonas* sp*.*	COT, OXA, AUG, ERY, TET, VAN	0.5	54.5
	*Aeromonas* sp*.*	COT, GEN, OXA, CAZ, AUG, ERY, TET, VAN	0.7	72.7
D	*Aeromonas* sp.	COT, OXA, CAZ, AUG, CIP, ERY, TET, VAN	0.7	72.7
E	*Pseudomonas* sp.	COT, GEN, OXA, AUG, ERY, TET, VAN	0.6	63.6
	*Aeromonas* sp*.*	OXA, CAZ, ERY	0.3	27.3
	*Pseudomonas* sp.	MEM, COT, OXA, AUG, ERY, TET, VAN	0.6	63.6
	*Aeromonas* sp*.*	OXA, CAZ	0.2	18.2

Key: B — Apitipiti 2; C — Apitipiti 3; D — Sogidi; E — Aba Apa Akinmorin; R — resistant; I — intermediate; S — sensitive and µ — microgram. IMI — Imipenem (10 µg); MEM — Meropenem (10 µg); COT — Cotrimoxazole (25 µg); GEN — Gentamicin (10 µg); CAZ — Ceftazidime (30 µg); AUG — Augmentin (20 µg); CIP — Ciprofloxacin (5 µg); ERY — Erythromycin (15 µg); VAN — Vancomycin (30 µg); TET — Tetracycline (30 µg); OXA — Oxacillin (1 µg); MAR — multi antibiotics resistance.

In this study, it was observed that one Gram-positive (*Corynebacterium* sp.) and one Gram-negative (*Aeromonas* sp.) species revealed the highest MAR of 90.9% resistance to the antibiotics tested. This corroborates the findings of Ayandele et al. (2019) in Nigeria who reported the multidrug-resistant trait of *Corynebacterium* species isolated from a close by dam to where the current study was carried out. Furthermore, in Brazil, Conte et al. (2021) published the multidrug resistance of *Aeromonas* species isolated from aquatic environments. The observed high frequency of bacterial resistance in this study is of huge health concern. This phenomenon may result in the therapeutic failure of the river fauna population. In addition, the human health of the community may be endangered by being at risk of infection from pathogens animals, coupled with the possibility of plasmid transfer of resistance to human pathogenic bacteria (Nwobodo et al., 2022).

The occurrence of antibiotic-resistant bacteria in the rivers sampled for this study reveals that antibiotics may be indiscriminately used by members of the communities. The resistance pattern by the isolated bacteria to most of the antibiotics tested may likely be through the discharge of antibiotics in considerable amounts via human waste (Dahunsi et al., 2014). Usually, indigenous bacteria in their natural environments develop resistance to antibiotics mostly when they persist in such environments; hence, this supports bacterial antibiotic resistance selection. This might also be due to the ineffectual regulation of using antibiotics to treat gastrointestinal infections, which results in fewer alternative treatments (Delgado-Gardea et al., 2016). However, grave human health implications have been associated with antibiotic resistance. It is estimated that by 2050, antibiotic resistance (AR) may account for about 10 million deaths and a huge financial burden of around US$100 trillion (O’Neill, 2016). The transfer of ARBs and ARGs from diverse water sources has been found to affect human health (Serwecińska, 2020). ARBs and ARGs residues could negatively influence human health through the consumption of contaminated water and agricultural products, resulting in the development of diverse health challenges such as allergic reactions and cancers (Serwecińska, 2020).

In addition, antibiotic resistance could be transferred from the environment to drinking water sources, thus posing serious human health threats such as increased mortality and prolonged morbidity (Chamosa et al., 2017). Antibiotic resistance may result in enhanced virulence, pathogenicity, disease outbreaks and transmission, leading to prolonged morbidity and hospitalisation, and even mortality (Ashbolt, 2013; Berendonk et al., 2015).

Environmental factors play a crucial role in the development and spread of antibiotic-resistant bacteria (ARB). Water used for agriculture, recreation, drinking and other domestic purposes are risk sources to humans. Therefore, the frequent assessment of the antimicrobial resistance patterns in potable water and its sources is paramount for monitoring the spread, which can assist in the development of preventive strategies (Ateba et al., 2020). Antibiotic resistance increases patient mortality and morbidity as well as increases the financial burden of disease especially in low-income countries like Nigeria. Hence, proper programmes to monitor antimicrobial usage and resistance in bacteria from aquatic environments are encouraged for implementation in Nigeria. This is essential for the fact that acquired bacterial resistance to antibiotics is prevalent as seen in this study, which is usually due to the misuse and abuse of antibiotics (Hoorzook et al., 2021).

## Conclusion

The physicochemical parameters, microbial identities and diversity as well as the antibiotic resistance profile of five rivers were assessed in this study. Each of the five rivers examined in this study has its peculiarity as regards the parameters tested in evaluating the standard quality of the water samples. The river sampled revealed some level of pollution due to recorded high microbial load and prevalence of multidrug-resistant bacteria. The pH and temperature were of optimum values within the WHO and NIS permissible limits. On the other hand, EC, turbidity, COD and BOD exceeded the permissible limits. Generally, most heavy metals tested — Cu, Cd, Cr, Mn, Zn, Ni and Pb — were within acceptable drinking limits across the five rivers except Fe. There were indications of interactions among the physical, chemical and biological parameters. With regard to microbial quality, all the river sampled were found unfit and not potable according to the WHO standard. This study also recorded one of the rivers having as high as 90.9% MDR prevalence. This may pose a human health threat due to vertical/horizontal gene transfer. Overall, this study reveals that these rivers are unfit for consumption/domestic or recreation purposes. Proper treatment by chlorination and ultra-violet treatment are therefore suggested if these water sources are to be used for human consumption. This study is only preliminary; extensive research into each of these rivers and associated environmental conditions as well as investigation into the possible long-term risks of water borne diseases via the ingestion of these drug-resistant bacteria is advocated.

## Limitations of the study

Firstly, this study was conducted only during the dry season. This might not capture the full range of water quality variations throughout the year, as wet seasons could lead to different pollutant levels and microbial profiles. Secondly, the study cannot be generalised as it focused on rivers within Oyo town, Nigeria. This might not represent the broader water quality situation in Nigeria or other parts of the world, as there could be significant variations across different regions.

## Supplementary Information

Below is the link to the electronic supplementary material.

Supplementary file1 (DOCX 16.9 KB)

## Data Availability

No datasets were generated or analysed during the current study.
