# Supplementary material for: Bacterial isolates from drinking water river sources exhibit multi-drug resistant trait
Source: Environ Monit Assess. 2024 Oct 15;196(11):1054. doi: 10.1007/s10661-024-13117-9 (PMC11480157; doi:10.1007/s10661-024-13117-9)
Supplement: Supplementary file 1 — Supplementary file1 (DOCX 16.9 KB) [file 10661_2024_13117_MOESM1_ESM.docx]

**Supplementary information**

**Table 1: Pearson’s correlation matrix of physical, chemical, biological parameters and total heterotrophic bacterial counts in sampled rivers**

|  | **Temperature** | **pH** | **EC** | **Chloride** | **Sulphate** | **Phosphate** | **Turbidity** | **Nitrate** | **COD** | **BOD** | **Fluoride** | **THB** |
| --- | --- | --- | --- | --- | --- | --- | --- | --- | --- | --- | --- | --- |
| Temperature | 1 |  |  |  |  |  |  |  |  |  |  |  |
| pH | 0.83 | 1 |  |  |  |  |  |  |  |  |  |  |
| EC | 0.16 | 0.39 | 1 |  |  |  |  |  |  |  |  |  |
| Chloride | 0.32 | 0.51 | **0.98**** | 1 |  |  |  |  |  |  |  |  |
| Sulphate | **0.93*** | **0.96**** | 0.21 | 0.35 | 1 |  |  |  |  |  |  |  |
| Phosphate | 0.65 | 0.43 | 0.62 | 0.73 | 0.44 | 1 |  |  |  |  |  |  |
| Turbidity | **0.91*** | 0.77 | -0.08 | 0.03 | **0.90*** | 0.32 | 1 |  |  |  |  |  |
| Nitrate | -0.67 | -0.71 | -0.11 | -0.30 | -0.70 | -0.44 | -0.45 | 1 |  |  |  |  |
| COD | 0.18 | -0.18 | **-0.92*** | **-0.86*** | 0.06 | -0.32 | 0.40 | -0.01 | 1 |  |  |  |
| BOD | 0.75 | 0.30 | -0.43 | -0.27 | 0.53 | 0.39 | 0.73 | -0.46 | 0.73 | 1 |  |  |
| Fluoride | 0.80 | 0.57 | -0.43 | -0.26 | 0.75 | 0.19 | 0.84 | -0.65 | 0.67 | **0.89*** | 1 |  |
| THB | -0.83 | -0.81 | 0.18 | 0.01 | **-0.90*** | -0.20 | -0.84 | 0.80 | -0.38 | -0.67 | **-0.92*** | 1 |

*, *p* < 0.05; **, *p* < 0.01

**Table 2: Relationships between heavy metals and total bacterial counts in sampled rivers**

|  | **Iron** | **Copper** | **Zinc** | **Lead** | **Nickel** | **Manganese** | **THB** |
| --- | --- | --- | --- | --- | --- | --- | --- |
| Iron | 1 |  |  |  |  |  |  |
| Copper | -0.10 | 1 |  |  |  |  |  |
| Zinc | -0.24 | -0.30 | 1 |  |  |  |  |
| Lead | **0.93*** | -0.19 | -0.09 | 1 |  |  |  |
| Nickel | -0.51 | -0.25 | 0.72 | -0.20 | 1 |  |  |
| Manganese | **0.91*** | -0.04 | 0.14 | 0.85 | -0.33 | 1 |  |
| THB | -0.64 | 0.21 | -0.06 | -0.40 | 0.62 | -0.73 | 1 |

*, *P* < 0.05

**Table 3: Antibiotics Tested and Resistance Cut off Values Used**

| **Antibiotic** | **Abbreviation** | **Concentration (µg/mL)** | **Susceptible (mm)** | **Intermediate (mm)** | **Resistant (mm)** | **Sensitive No. (%)** | **Intermediate No. (%)** | **Resistance No. (%)** |
| --- | --- | --- | --- | --- | --- | --- | --- | --- |
| Imipenem | IMI | 10 | ≥23 | 17-22 | ≤16 | 84.8 | 9.1 | 6.1 |
| Meropenem | MEM | 10 | ≥22 | 17-21 | ≤16 | 78.8 | 3 | 18.2 |
| Cotrimoxazole | COT | 25 | ≥19 | 16-18 | ≤15 | 48.5 | 6 | 45.5 |
| Gentamicin | GEN | 10 | ≥15 | 12-14 | ≤11 | 81.8 | 6.1 | 12.1 |
| Ceftazidime | CAZ | 30 | ≥22 | 17-21 | ≤16 | 18.2 | 3 | 78.8 |
| Augmentin | AUG | 20 | ≥22 | 17-21 | ≤16 | 57.6 | 3 | 39.4 |
| Ciprofloxacin | CIP | 5 | ≥23 | 17-22 | ≤16 | 72.7 | 6.1 | 21.2 |
| Erythromycin | ERY | 15 | ≥22 | 17-21 | ≤16 | 21.2 | 3 | 75.8 |
| Vancomycin | VAN | 30 | ≥30 | 23-29 | ≤22 | 24.2 | 18.2 | 57.6 |
| Oxacillin | OXA | 1 | ≥29 | 20-28 | ≤19 | 8.6 | 8.6 | 82.8 |
| Chloramphenicol | CHL | 12.5 | ≥25 | 17-24 | ≤16 | 23.8 | 0 | 76.2 |
| Tetracycline | TET | 30 | ≥22 | 17-21 | ≤16 | 27.3 | 0 | 72.7 |
